# The Effects of Agricultural and Urban Land Use on Drinking Water Treatment Costs: An Analysis of United States Community Water Systems

**DOI:** 10.1142/s2382624x20500083

**Published:** 2020-10-14

**Authors:** James I. Price, Matthew T. Heberling

**Affiliations:** * US Environmental Protection Agency Office of Research and Development Cincinnati, OH 45268, USA; † School of Freshwater Sciences University of Wisconsin — Milwaukee Milwaukee, WI 53204, USA

**Keywords:** Community water system, avoid treatment cost, water quality, land use, source water protection

## Abstract

For community water providers, safeguarding source waters from contamination offers an additional barrier of protection and a potential means of avoiding in-plant treatment costs. Whether source water protection efforts are cost-effective relative to in-plant treatment requires hydrologic, geologic, and climatologic knowledge of source watersheds, as well as an understanding of how changes in source water quality affect treatment costs. Quantitative evidence on the latter relationship is limited. This study estimates separate hedonic cost functions for water systems that primarily use surface water sources and those that primarily use groundwater sources using a database of United States (US) Community Water Systems. Cost functions relate annual variable treatment cost to production, factor input prices, capital stock, and source water quality, as proxied by land use within various ex-ante defined contributing areas (i.e., surrounding land areas affecting source water quality). For surface water systems, a 1% increase in urban land relative to forestland is correlated with a 0.13% increase in annual variable treatment costs. In this analysis, the relationship between costs and agricultural land is not statistically significant. Conversely, for groundwater systems, a 1% increase in agricultural land relative to forestland is correlated with a 0.24% increase in costs, whereas in this analysis the relationship between costs and urban land is not statistically significant. The cost-effectiveness of forestland preservation, based on sample means, varies considerably with the size of the contributing area, with no clear indication as to whether preservation is more likely to be cost-effective for surface water or groundwater systems.

## 1. Introduction

Drinking water providers often face declines in source water quality due to long-term changes in watershed conditions, prominent drivers of which include land use conversion, urban and agricultural runoff, and wildfire events (Gartner *et al*. 2013; Postel and Mastny 2005). In response, water providers can modify or augment existing treatment processes to maintain compliance with regulatory standards and performance objectives. They can also undertake actions that protect source water *in situ*, such as land acquisition and management, incentivizing landowners to adopt best management practices, and public education campaigns (Bennett *et al*. 2014; Gartner *et al*. 2013; Carpe Diem West 2011). Accordingly, a key question for water providers, as well as regulators, is whether source water protection (SWP) is cost-effective relative to in-plant treatment options. Decision-makers need to understand physical and chemical processes, such as watershed hydrology, sediment dynamics, and ecology that affect water quality to answer this question, as well as how changes in source water quality affect treatment costs. Evidence-based information on the latter is limited and has been cited as a barrier to upscaling watershed investments (Bennett *et al*. 2014; Gartner *et al*. 2013; Postel and Thompson 2005).

Cost functions can be used to relate treatment costs to source water conditions. Extant studies predominantly find a positive correlation between treatment costs and source water contamination (Price and Heberling 2018). Turbidity is the most commonly used water quality measure in these studies; however, total organic carbon, pH, nitrates, and calcium carbonate are also used (Price and Heberling 2018). For turbidity, results show that a 1% decline in source water quality leads to a 0.07–0.3% increase in costs.^1^

In other studies, land use near surface water intakes or wellheads is employed as a proxy for source water quality. Causal relationships between broadly defined land use types and water quality are well established, where agriculture and urban land are correlated with lower quality surface water and groundwater relative to forestland (Lerner and Harris 2009; Baker 2003; Dudley and Stolton 2003). For United States (US) water treatment systems, Freeman *et al*. (2008) and Ernst *et al*. (2004), both non-peer-reviewed reports published by The Trust for Public Land, find that increasing forestland by 1% relative to non-forestland within source watersheds reduces variable treatment costs by 0.38% and 0.83% per year, respectively. Warziniack *et al*. (2017) estimate two independent equations: one that evaluates the effect of turbidity on treatment costs and another that evaluates the effect of forestland on source water turbidity. Exploiting the recursive relationship between equations suggests that increasing forestland by 1% reduces variable treatment costs by 0.32% per year. Likewise, Forster and Murray (2007) evaluate the effect of turbidity on treatment costs and, in an independent equation, the effect of various agricultural tillage practices on source water turbidity. Results indicate that shifting from ridge and mulch tilled land to tilled land with less than 15% residual increases treatment costs by 0.4%. Shifting to non-agricultural land also leads to higher costs but shifts to untilled land and tilled land with 15–30% residual have no statistically significant effect on costs.

Abildtrup *et al*. (2013) and Fiquepron *et al*. (2013) evaluate the effects of land use on water rates in France, which are assumed to represent long-run average treatment costs. Both studies find forestland to be correlated with lower costs relative to agricultural and urban land. For water providers in Portugal, Lopes *et al*. (2018) find forestland has no statistically significant effect on variable treatment costs for providers relying entirely on surface water sources. Forestland, however, is negatively correlated with costs for providers with at least one groundwater source. Vincent *et al*. (2016) distinguished between virgin and logged forestland in their analysis of Malaysian treatment plants. Findings indicate that having 1% more virgin forestland relative to non-forestland within source watersheds reduces variable treatment costs by 0.47% per year. The corresponding reduction for logged forestland is 0.32%. Singh and Mishra (2014) estimate multiple independent equations, similar to Warziniack *et al*. (2017) and Forster and Murray (2007), for a single water treatment plant in India. Increasing forestland by 1% is shown to reduce variable costs by 1.58% per year.

The present study extends this literature using a database of US Community Water Systems (CWSs) that contains information on production, expenditures, treatment processes, and landscape characteristics (e.g., land slope, precipitation, land use) near surface water intakes and wellheads.^2^ We estimate separate cost functions for CWSs that primarily use surface water sources and those that primarily use groundwater sources and subsequently calculate the benefits (i.e., avoided treatment costs) associated with lower levels of agriculture and urban land use. The cost functions are rooted in economic theory but are informed by hydrologic and ecologic considerations. Results offer insight into the roles of environmental factors in potable water production and how roles differ across predominantly surface water and groundwater systems. Empirical evidence on these differences has been identified as a key gap in the SWP literature (Price and Heberling 2018).

## 2. Empirical Model

This analysis uses a hedonic cost function to model variable treatment costs. According to economic theory, variable costs are a function of output quantity, factor input prices, and quasi-fixed capital stock (Varian 1992). The hedonic cost function extends this framework by allowing costs to depend on characteristics of production inputs and outputs (Holmes 1988). We specify a Cobb–Douglas type model, in which in addition to those factors dictated by theory, costs are a function of source water quality, which we proxy by landscape characteristics near surface water intakes and wellheads. The model takes the form

$$c_{i}=\alpha y_{i}^{\beta_{y}}k_{i}^{\beta_{k}}\left(\prod_{h}P_{hi}^{\beta_{ph}}\right)\left(\prod_{m}Q_{mi}^{\beta_{qm}}\right)\exp\left(\sum_{j}\beta_{sj}S_{ji}+\varepsilon_{i}\right),$$

where *c* represents the variable treatment costs at CWS *i, y* the output quantity, *k* the capital stock, *P* a vector of factor input prices, *Q* a vector of landscape characteristics, *S* a vector of other treatment process attributes, and ε an error term. The *α* and *β* terms are parameters to be estimated. Landscape characteristics include average precipitation, average land slope, and the fraction of various land uses (e.g., cropland, pasture, urban) within specified areas near intakes and wellheads. Multiple spatial scales, as described later, are used to evaluate these characteristics.

The hedonic cost function is predicated on cost-minimizing behavior, whereby CWSs minimize expenditures by selecting the optimal quantity of variable inputs needed to produce some level of output. A common, sometimes implicit, assumption is that a CWS’s output is exogenously determined because of regulatory constraints that limit control over production levels, such as caps on revenue and requirements to meet customer demand (Mosheim and Ribaudo 2017; Price *et al*. 2017; Warziniack *et al*. 2017; Horn 2011; Holmes 1988). Torres and Paul (2006), however, argue that output may be endogenous because optimizing decisions are based on expected water demands — over which CWSs exercise control. They address endogeneity using a full information maximum likelihood approach. Other studies, including Abildtrup *et al*. (2013), Destandau and Garcia (2014), and Lopes *et al*. (2018), address endogeneity using instrumental variable methods. We evaluate endogeneity using a two-stage least-squares approach, where, like Destandau and Garcia (2014) and Lopes *et al*. (2018), the number of residents served by CWSs is used as an instrument for production output. We find residential population to be a valid and strong instrument, whereas results from Wu–Hausman tests confirm the presence of endogeneity. The two-stage least-squares model is thus the preferred estimator. We assume that land use variables are exogenous. Although CWSs exert influence over land use when selecting intake and well locations and through land management programs, any such decisions are largely fixed in the short run.

We anticipate that the relationships between treatment costs and its predictors will differ systematically depending on whether a CWS primarily uses surface water or groundwater sources. Evidence in support of this view is found in cost function analyses (Lopes *et al*. 2018), as well as studies linking land use to source water quality (Hurley and Mazumder 2013; Johnson and Belitz 2009). We therefore estimate separate models for CWSs that exclusively or primarily use surface water sources (hereafter surface water systems) and those that exclusively or primarily use groundwater sources (hereafter groundwater systems). The US Environmental Protection Agency (USEPA) classifies CWSs by their predominant water source type. Most CWSs classified as surface water systems (74%) exclusively use surface water sources; likewise, most CWSs classified as groundwater systems (96%) exclusively use groundwater sources (USEPA 2009). Among the minority of surface water systems that do not solely use surface water, 82% of water, on average, is drawn from surface water sources (USEPA 2009). For groundwater systems that do not solely use groundwater sources, 78% of water, on average, is drawn from groundwater sources (USEPA 2009). We use the USEPA’s classification to identify surface water and groundwater systems for this analysis. Landscape characteristics (vector Q) pertain to specified areas near surface water intakes for surface water systems and areas near wellheads for groundwater systems. For CWSs that use both surface water and groundwater sources, only landscape characteristics related to the primary water source type are included in the model. We believe that this will have limited effect on results because the vast majority of CWSs has an overwhelmingly dominant source water type.

For model estimation, we use the logged form of the hedonic cost function with a two-stage least-squares estimator and bootstrapped standard errors based on 1,000 replications. We impose linear homogeneity on factor input prices, a feature consistent with a well-behaved cost function (Varian 1992), by constraining the sum of parameters in vector *β*_*ph*_ to unity.^3^ Following Vincent *et al*. (2016), we modify land use variables by replacing *Q*_*mi*_ with 1 + *Q*_*mi*_ prior to taking logs. Without this modification, observations with a zero value would be undefined. The associated elasticities of cost are determined by ${\hat{\beta }}_{qm}\times {\bar{Q}}_{m}/\left(1+{\bar{Q}}_{m}\right)$, where $\hat{\beta }$ is the estimated coefficient and ${\bar{Q}}_{m}$ is the mean value of the land use variable of interest. The resulting elasticities are the percentage change in treatment cost resulting from a 1% change in the land use variable within the specified area.^4^ Standard errors for the elasticities are calculated using the delta method.

## 3. Data

### 3.1. Treatment costs, factor prices, and cws characteristics

Data used in this analysis are largely obtained from two USEPA surveys: the 2006 Community Water System Survey (CWSS) and the Water Treatment Plant Questionnaire (WTPQ). These surveys are the most recent USEPA surveys to elicit the information needed to estimate a hedonic cost function and they have not, to the best of our knowledge, been used before for a comparable analysis. The CWSS was designed to support a variety of regulatory, policy, and compliance analyses. It contains records for 1,314 CWSs, which were sampled from US CWSs using a stratified random sampling procedure based on source water type and population served (USEPA 2009). To maintain data accuracy and sample representativeness, the USEPA sent water systems experts to collect data from CWSs serving populations less than 3,300 (USEPA 2009).^5^ Without assistance, some of these systems may not have had the capacity to respond to some survey questions. The WTPQ was designed specifically to inform effluent guidelines regulations and, thus, collected detailed information on residuals generation, treatment, and disposal. It contains records for 378 randomly sampled CWSs operating treatment plants that generate residuals and serve populations greater than 10,000. Both surveys elicited operational and financial information for the 2006 calendar year. While not identical, many survey questions were sufficiently alike to justify pooling responses; thus, we combine the CWSS and WTPQ samples for a total of 1,692 records. Several records were subsequently excluded from the analysis as a result of the data cleaning process.

Survey responses were evaluated to ensure compatibility between operational and financial information. First, we exclude from the analysis observations where financial information pertains to multiple CWSs, but operational information pertains to a single CWS. This occurred in some instances when the water provider, typically a private entity, operates multiple CWSs. Secondly, we exclude observations where financial information pertains to both water supply and wastewater services. Thirdly, we exclude observations where financial information relates to a period other than the 2006 calendar year (i.e., a fiscal year or alternate calendar year), apart from a few instances in which both operational and financial information are reported for the same alternate period. In these cases, we inflate dollar values to 2006 prices using the All Commodities Producer Price Index (US Bureau of Labor Statistics 2018) and retain the observation for analysis.

We also carefully reviewed open-ended comments on the CWSS and WTPQ, subsequently adjusting data to correct errors and flagging areas of concern (e.g., data abnormalities, inconsistencies). Based on the latter group, we exclude observations where key relationships are not accurately preserved due to, for example, double counting, partial reporting of values, or atypical events in the 2006 calendar year. Open-ended comments also made clear the ambiguity of some survey questions. Numerous water providers expressed uncertainty about how to define an employee, classify employees by sub-category (e.g., engineer, administration), and report employee compensation — suggesting wage rate data, which are calculated from this information, may be noisy. To a lesser extent, water providers expressed uncertainty about how to report certain expenditures and funding sources. After removing observations with missing values and outliers in wage rates and per unit cost of production based on visual inspection of the data, the surface water and groundwater datasets contain 296 and 200 observations, respectively.^6^

For the cost function analysis, variable costs (*VarCost*) are defined as the sum of routine expenses for labor, chemicals, power, materials and supplies, contractor services, and water purchases. Security-related costs, although mostly fixed rather than variable, are also included. Production output (*WatVol*) is the total volume of water in million gallons delivered to residential customers, non-residential customers, and other CWSs. We control for the fraction of production sold to other CWSs (*Sold*). This water may have lower delivery costs than water supplied through municipal distribution networks, and it may, if only partially treated, be associated with lower variable costs. Factor input prices consist of the average electricity rate (*ElecRate*) and average wage rate of full-time employees (*WageRate*). Electricity rates — not available in the CWSS or WTPQ — are obtained from the US Energy Information Administration’s Electricity Data Browser (US Energy Information Administration 2016) and matched to CWSs by US state. A suitable source for chemical prices could not be identified; thus, these prices, as well as those for other inputs, are subsumed into the model’s error term. The use of state-level electricity rates introduces measurement error into the model and is a potential source of bias, as is the omission of chemical prices. The measurement error leads to attenuation bias in the electricity rate coefficient and possible bias in the other coefficients, depending on the degree of measurement error and correlations between independent variables (Oberski and Satorra 2013).

Following Mosheim and Ribaudo (2017) and Mosheim (2006), we calculate capital stock (*CapStock*) to be the ratio of operating profit to the opportunity cost of capital. Operating profit is calculated as the difference between a CWSs revenue and variable costs. The opportunity cost of capital is calculated as the rate of depreciation plus a weighted average of the cost of debt and the cost of equity, where weights are determined by the proportion of capital expenditures, as reported in the CWSS and WTPQ, funded by each source in the past five years.^7^ Under this approach, CWSs are assumed to earn a normal profit — as would be the case with average cost pricing — and to minimize long-run costs by optimally choosing quantities of all inputs. The measure of capital stock will therefore be biased to the extent that these assumptions are violated (Mosheim and Ribaudo 2017). In accordance with economic theory, variable treatment costs are expected to be non-increasing with respect to capital stock.

We also control for CWSs’ primary treatment technology. CWSs are classified as having conventional filtration (*ConFilt*), direct filtration or disinfection (*DirFilt*), or other technologies (*OthTech*). Conventional filtration entails coagulation, flocculation, sedimentation, filtration, and disinfection treatment processes, whereas direct filtration or disinfection employs subsets of these processes (Crittenden *et al*. 2012). Other technologies include membrane filtration, aeration, and ion exchange, as well as any undefined treatment methods. We recognize that there is an endogenous relationship between variable treatment costs and treatment technology that may bias parameter estimates, but, as in Price *et al*. (2017) and Warziniack *et al*. (2017), we include technology indicators in some model specifications to address potential omitted variable bias. Finally, the cost function includes fixed effects for USEPA regions (*RegA-E*) and, for the surface water model, an indicator for whether source water is obtained from a lake or reservoir as opposed to a river (*Reservoir*). In the CWSS and WTPQ, information on treatment technology and source water type (i.e., reservoir, river) are recorded for treatment plants rather than CWSs. When CWSs operate multiple plants, we determine their categorization based on how most water is processed and extracted.

Descriptive statistics for the variables defined above are reported in Table 1. Of the 296 CWSs in the surface water dataset, 46% completed the CWSS but not the WTQP, 35% completed the WTPQ but not the CWSS, and 19% completed both surveys. Average production for surface water CWSs was 6,988 million gallons in 2006, which is substantially more than the national average of 3,908 million gallons for all surface water systems as reported in the 2006 CWSS report (USEPA 2009). Of the 200 CWSs in the groundwater dataset, 89% completed the CWSS, 8.5% completed the WTPQ, and 2.5% completed both surveys. Average production for groundwater CWSs was 1,465 million gallons in 2006. By comparison, average production, as reported in the 2006 CWSS, during the same period for all groundwater systems was 1,729 million gallons (USEPA 2009). A breakdown of dataset observations by their source (i.e., CWSS or WTPQ) is available in the Supplementary Material, as is additional information regarding the representativeness of the surface water and groundwater samples. Surface water and groundwater systems have similar average wage rates, but surface water systems are more likely to use conventional treatment methods and to sell water to other CWSs.

### 3.2. Land use, precipitation, and slope

**Figure 1.**
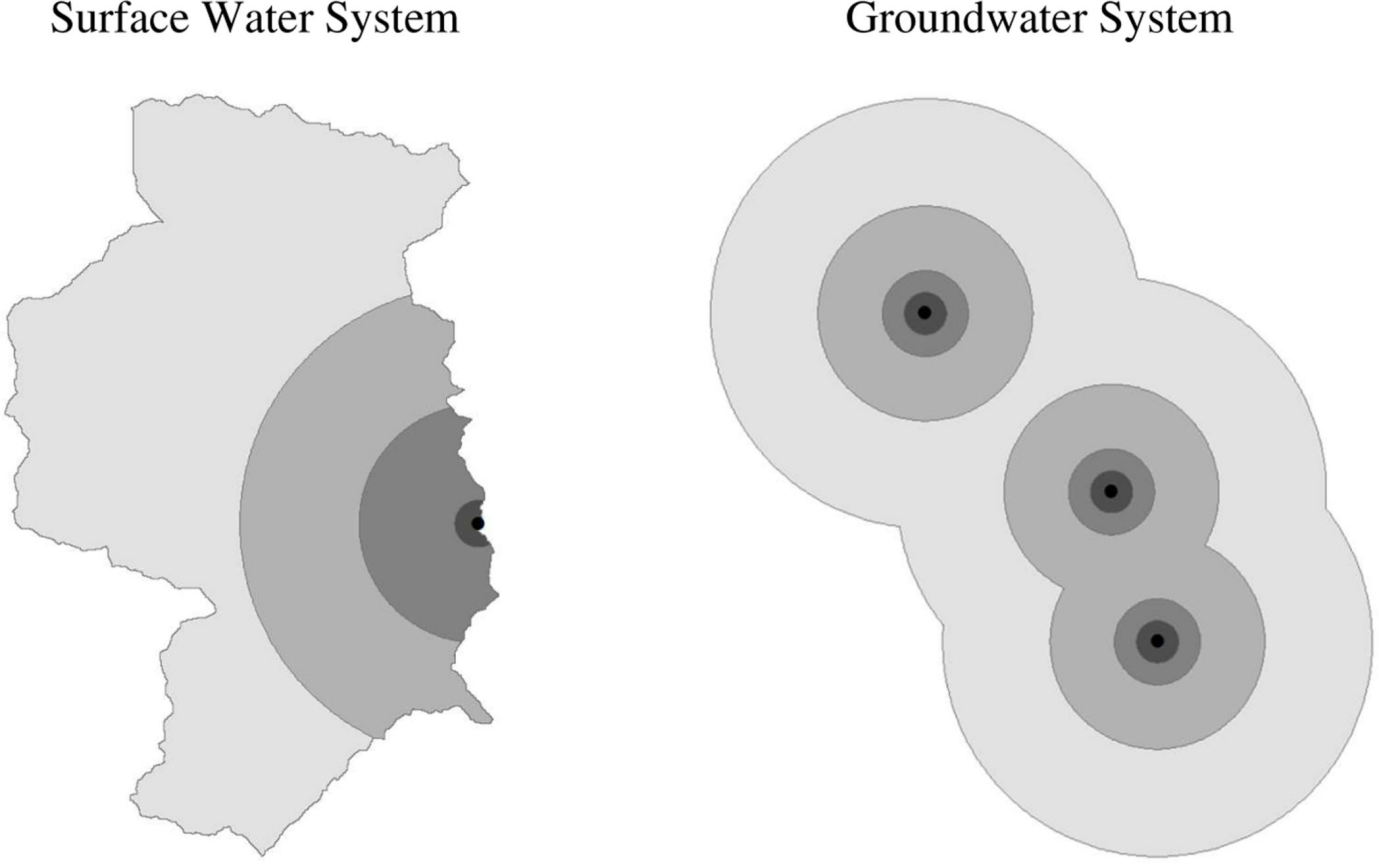
Example Contributing Land Use Areas *Notes*: Black dots represent either surface water intakes or wellheads for a single CWS. Shaded areas represent contributing areas. For surface water systems, these contributing areas are defined as upslope land within 1, 5, and 10 km of the intake, as well as the entire upslope drainage basin. For groundwater systems, contributing areas are defined as land within 0.1, 0.5, 1, and 5 km of wellheads. Larger contributing areas are inclusive of smaller areas. When water systems have multiple intakes or well-heads, contributing areas are created by delineating an area for each intake or wellhead and then merging these areas; thus, there is a single contributing area at each spatial scale for each water system.

The USEPA Office of Water maintains a database of point coordinates for surface water intakes and wells used for public water supply. We employ land use near these coordinates as a proxy for source water quality. But, given differences between surface water and groundwater hydrology, we define different contributing areas (i.e., land areas affecting source water quality) for surface water intakes and wells. For intakes, we rely on geospatial data developed by Wickham *et al*. (2011). These data consist of drainage basins for 5,265 intakes listed in the USEPA Office of Water database, with each basin representing the upslope area that contributes water flow to an intake’s location. From these data, we delineate, in addition to the entire basin, upslope areas within 1, 5, and 10km of each intake (see Figure 1).^8^ For CWSs with multiple intakes, the resulting areas are merged to create a single contributing area at each spatial scale. Finally, using the National Land Cover Database (NLCD) 2006 (Homer *et al*. 2015), we calculate the fraction of each contributing area classified as developed (*Urban*), agriculture (*Ag*), other land (*OthLand*), and forestland (*Forest*), where agricultural is decomposed into cropland (*Crop*) and pasture (*Pasture*) in some model specifications. The effects of cropland and pasture on water quality may differ due to differences in erosion rates, fertilizer application (e.g., type, intensity, timing of use), and runoff patterns, with cropland likely contributing to greater sediment and nutrient loading (Rajib *et al*. 2016; Harmel *et al*. 2006; Hubbard *et al*. 2004). The other land category (*OthLand*) comprises barren land, shrubland, grassland, wetland, and open water classifications, which were combined for the purpose of this analysis.

Although contributing areas for wells, like those for intakes, extend upslope, they often require detailed groundwater flow models to define their size and shape (Johnson and Belitz 2009). In the absence of these models, studies evaluating correlations between land use and groundwater quality have primarily used circular buffers, with radii ranging from 0.3km to 3.2km, to represent contributing area (Fram and Belitz 2011; Squillace and Moran 2007; An *et al*. 2005; Gardner and Vogel 2005; Moran *et al*. 2005; Aelion and Conte 2004; Worrall and Kolpin 2004). In a study of California supply wells, Johnson and Belitz (2009) estimate the relationship between land use and volatile organic compounds using contributing areas of various shapes and sizes. They conclude that 0.5 km circular buffers adequately reflect the land use patterns affecting water quality, even though other size circular buffers and wedge-shaped areas that are oriented upslope performed slightly better. In keeping with these findings and extant literature, we delineate circular buffers around wellheads with radii of 0.1, 0.5, 1, and 5km (see Figure 1). As before, these areas are merged if they belong to the same CWS and spatial scale, and land use fractions are calculated.

We also incorporate average annual precipitation and land slope within contributing areas into the cost function. Both variables have previously been associated with source water quality and water treatment costs (Lopes *et al*. 2018; Vincent *et al*. 2016; Singh and Mishra 2014; Dearmont *et al*. 1998). Average precipitation calculations are based on 30-year climate normal data available from the Parameter-Elevation Regressions on Independent Slopes Model (PRISM) Climate Group (Daly 2013). Average land slope calculations are based on 100-m resolution data derived from the US Geological Survey’s National Elevation Dataset (Gesch *et al*. 2002), where slope is determined by the maximum rate of change between a cell and its neighbors.

Descriptive statistics for land use, precipitation, and slope variables are presented in Tables 2 and 3, for surface water and groundwater samples, respectively. On average, the fraction of forestland, cropland, and pasture increases with the size of the contributing area, whereas the fraction of urban land decreases. For surface water, average values range from 0.04 to 0.09 for cropland, 0.06 to 0.10 for pasture, and 0.1 to 0.21 for urban land. The corresponding ranges for groundwater are 0.12–0.2, 0.1–0.11, and 0.19–0.37. Hurley and Mazumder (2013) find evidence that key source water quality parameters (e.g., turbidity, TOC, *Escherichia coli*) are influenced at different spatial scales of land use; thus, we have no prior expectation as to the relative effect of different size contributing areas on cost.

## 4. Results

Cost function results for the surface water sample are reported in Table 4.^9^ Specifically, two model specifications are reported for the 5, 10, and full-basin areas, where specifications differ in whether the fraction of land classified as agricultural is defined in the aggregate (Model 1) or decomposed into cropland and pasture (Model 2). Results for the 1 km contributing area, as well as alternate model specifications at all spatial scales, are provided in the Supplementary Material. All land use variables in the 1km contributing area models are not statistically significant.

Consistent with economic theory, *WatVol*, *WageRate*, and *ElecRate* are positively correlated with variable treatment cost. For *WatVol*, estimated parameters indicate that a 1% increase in production leads to a 1% increase in cost, which is similar, albeit somewhat larger, to findings from other analyses (Lopes *et al*. 2018; Mosheim and Ribaudo 2017; Holmes 1988) and implies the presence of constant short-run returns to scale (i.e., average cost does not change as production increases). Wald tests confirm that the coefficient for *WatVol* is not significantly different from one in all model specifications. *CapStock* is negatively correlated with cost. This result, consistent with economic theory, implies that a marginal increase in capital stock reduces variable treatment costs. The coefficient for *Sold* is also negative, indicating that CWSs selling a larger fraction of their production to other water systems have, all else equal, lower costs. The coefficient for *Reservoir* is not statistically significant.

We find that relationships between treatment cost and landscape characteristics differ across spatial scales, with estimated effect sizes mostly declining as the contributing area increases. *Precip* and *Slope* are positively correlated with cost in the 5 km and 10 km contributing area models — consistent with the notion that watersheds with greater rainfall and steeper slopes have lower surface water quality due to increased particulate transport caused by higher levels of overland runoff, runoff velocity, and soil instability (Lintern *et al*. 2018). They are not statistically significant in the full-basin model, implying that the importance of these factors to source water quality diminishes with their distance from the intake. *Forest* is excluded from the estimated models to avoid multicollinearity and thus serves as the reference land use category.^10^ Relative to *Forest*, *Urban* is positively correlated with cost, although, as with *Precip* and *Slope*, the coefficient is not significant in the full-basin model. Agricultural land use variables and *OthLand* are not statistically significant for any spatial scales or model specifications. The land use elasticities of cost, reported in Table 5, indicate that a 1% increase in urban land within the 5km and 10km contributing areas, and a corresponding decrease in forestland, leads to a 0.12% and 0.13% increase in cost, respectively. A 1% increase in urban land within the entire drainage basin leads to a 0.08% increase in treatment costs, although this value is not significant at standard levels.

Cost function results for the groundwater sample, using the 0.5, 1, and 2.5km contributing areas, are reported in Table 6. Results for the 5km contributing area are provided in the Supplementary Material. As before, *WatVol* is positively correlated with variable treatment cost, and the magnitude of the estimated parameter implies the presence of constant returns to scale. This finding is confirmed by Wald tests showing that the coefficient on *WatVol* is not significantly different from one. *ElecRate* is positively correlated with cost, but, in contrast to the surface water sample, the coefficient on *WageRate* is not statistically significant, as are the coefficients on *CapStock* and *Sold*. Differences in results between CWS source types, particularly with regard to the relative importance of factor input prices, suggest that CWSs that primarily use surface water sources employ different production technologies than systems that primarily use groundwater sources — and support the decision to estimate separate models for each source type.

Relationships between treatment cost and landscape characteristics differ markedly between surface water and groundwater samples. In the groundwater sample, *Slope* exhibits a positive correlation with treatment cost that is largely constant across spatial scales, whereas *Precip* is not statistically significant. The mechanism whereby land slope affects groundwater quality is less evident than it is with surface water quality. A likely possibility is that steeper land surfaces are reflective of steeper subterranean hydraulic gradients (Fetter 2018), which, in turn, support faster groundwater flows, lower rates of attenuation, and higher concentrations of contaminants. Coefficients on *OthLand*, *Crop*, and *Urban* are not statistically significant. In contrast, *Ag* and *Pasture* are positively correlated with cost in the 0.5 km and 1 km contributing area models; they are not significant in the 5 km models. Estimated land use elasticities of cost, reported in Table 7, show that a 1% increase in agricultural land within the 0.1 km contributing area leads to a 0.24% increase in cost. Likewise, when agricultural land is disaggregated, 1% increases in cropland and pasture lead, respectively, to 0.13% and 0.14% increases in cost, although the former relationship is not significant at standard levels.

## 5. Discussion and Conclusion

SWP offers a means of lowering water-related health risks and avoiding treatment costs. These benefits are recognized in the USEPA’s Safe Drinking Water Act and some state water laws (California Water Code §108.5 2016; Tiemann 2014) and by the growing number of municipalities engaged in SWP activities (Bennett *et al*. 2014; Gartner *et al*. 2013; Carpe Diem West 2011; Herbert 2007; Dudley and Stolton 2003). However, decision-makers considering SWP often lack the quantitative estimates of its benefits needed to perform comprehensive cost-effectiveness or benefit–cost analyses. Benefit estimates pertaining to groundwater systems are especially limited (Price and Heberling 2018), despite that nearly three quarters of US CWSs rely primarily on groundwater sources (USEPA 2009).

This analysis extends the avoided treatment cost literature; it estimates the relationship between variable treatment costs and source water quality, as proxied by land use within various ex-ante defined contributing areas, using a database of US CWSs. For surface water systems, contributing areas are based on drainage basins that define the upslope area of each intake. The empirical model employed in the analysis is rooted in economic theory and controls for key determinants like output quantity, factor input prices, and quasi-fixed capital stock. Specification of the land use and other landscape characteristics are based on insights from the fields of hydrology, ecology, and economics. Results indicate that costs, relative to forestland, are positively correlated with urban land in the 5km and 10km contributing areas. Costs are not significantly correlated with agricultural land. For groundwater systems, contributing areas are delineated using circular buffers around wellheads. Costs are positively correlated with agricultural land in the 0.5km and 1km contributing areas, but they are not significantly correlated with urban area. Alternate model specifications, which disaggregate agricultural land, show that pasture, as opposed to cropland, is the primary driver of this relationship. We derive land use elasticities of cost from model estimates. These elasticities are consistent with those from similar studies, although direct comparisons are complicated by differences in land use categorization and model specification.

As in Lopes *et al*. (2018), we find substantial differences in how land use affects surface water and groundwater systems. On average, treatment costs for surface water systems included in this analysis are seemingly affected by source water contamination from urban areas but not agriculture. For these systems, the likely drivers of source water degradation are non-point source pollutants from storm-water runoff (e.g., sediments, nutrients, industrial chemicals) and point source pollutants originating with wastewater effluent and combined sewer overflows. Agriculture would have little impact if, among other possible explanations, intakes have been situated to avoid agricultural runoff or best-management practices on upslope agricultural land have been effective at reducing pollutant loads. Agriculture does affect surface water quality (USEPA 2002, 2017) though its impact on treatment costs is not significant in this analysis. Given increasing concerns about agriculture’s impact on water quality, it is possible that an analysis with more recent data would show a statistically significant relationship. Conversely, treatment costs for groundwater systems included in this analysis are affected by source water contamination from agriculture but not urban areas. The application of organic and inorganic nitrogen fertilizer to agricultural lands is a leading cause of groundwater pollution (Canter 1997) and is a likely driver of source water degradation for many groundwater sources. Urban-based contamination also affects groundwater quality (Shanahan and Jacobs 2007) though its impact on treatment costs is not significant in this analysis.

Whether SWP is cost-effective relative to in-plant treatment is highly contextual, being contingent on site-specific hydrologic, climatologic, and landscape characteristics, as well as the type and scale of protection activities under consideration. Among recent case studies, some conclude that the benefits of SWP exceed their costs (Kroeger *et al*. 2019; Hudak *et al*. 2013), whereas others reach the opposite determination (Moltz *et al*. 2018; Vincent *et al*. 2016; Heberling *et al*. 2015). Pyke *et al*. (2002) find that reducing agricultural runoff using tillage practices, but not riparian buffers, is cost-effective. The cross-section data used in this analysis are too coarse to account for localized conditions; nonetheless, results offer some insight into the cost-effectiveness of SWP. We calculate the benefits of preserving forestland using estimated elasticities and sample means. For surface water systems, preventing a 1% increase in urban land within the 5 km contributing area, and corresponding reduction in forestland, yields an annual benefit of $12,662 in avoided treatment costs. The corresponding benefit for the 10 km contributing area is $13,173. For groundwater systems, preventing a 1% increase in agricultural land within the 0.5km and 1km contributing areas would, on average, yield annual benefits of $4,150 and $4,465, respectively.

The cost of forestland preservation depends on the size of the area being protected and managed (McDonald and Shemie 2014). Using sample means, we calculate that surface water systems would need to preserve 0.064km^2^ of forestland in the 5km contributing area to realize the aforementioned benefit. The corresponding value for the 10km contributing area is 0.155km^2^. Similarly, groundwater systems would need to preserve 0.008km^2^ of forestland in the 0.5km contributing area and 0.027km^2^ of forestland in the 1km contributing area.^11^Accordingly, the largest benefit per amount of preserved forestland occurs to groundwater systems at the 0.5km contributing area (≈ $550,706/km^2^), followed by surface water systems at the 5km contributing area (≈ $196,618/km^2^), groundwater systems at the 1km contributing area (≈ $167,263/km^2^), and surface water systems at the 10km contributing area (≈ $84,867/km^2^). This finding reinforces the importance of scale in determining cost-effectiveness; namely, that the smaller the land area affecting source water quality the more likely SWP will be cost-effective relative to in-plant treatment (McDonald and Shemie 2014). It suggests the size of the land area affecting source water quality is a key factor in whether an SWP initiative should be undertaken, and the relevant spatial scale differs considerably between source types. Findings from this study, however, offer no clear indication as to whether forestland preservation is more likely to be cost-effective for surface water or groundwater systems. Additional information regarding the above calculations and values pertaining to the other contributing areas are provided in the Supplementary Material.

Continued research is needed to improve estimated relationships between treatment costs and source water quality. In this analysis, for instance, models estimated with the groundwater sample fail to control for key variables such as well depth and soil type and thus may be subject to omitted variable bias. Likewise, further research is needed to quantify relationships between treatment costs and key hydrologic, climatologic, and landscape characteristics, as well as to establish linkages between these variables and water quality parameters at the point of uptake. Estimating these relationships and linkages provide the translations needed to understand the cost-effectiveness of SWP programs. For example, management practices in the landscape improve source water quality which, in turn, reduce drinking water treatment costs. Comparing the costs of the management practices with the reduced treatment costs informs decision-makers about the cost-effectiveness of SWP programs. In this analysis, the land use proxies can help decision-makers better understand the landscape effects on treatment costs, but the specific pollutants or water quality parameters driving treatment costs are unknown. This limits policy to protecting land use and not land management practices that prevent or reduce pollutants. Making these connections will be crucial to designing effectual SWP programs and policy.

## Supplementary Material

1

## Figures and Tables

**Table 1.** Descriptive Statistics for Community Water System Characteristics

		Surface Water Facilities		Groundwater Facilities	
			
Variable	Definition	Mean	SD	Mean	SD
VarCost	Total 2006 variable costs (million USD)	10.21	21.27	1.85	8.50
WatVol	Total 2006 production (million gallons)	6,987.65	14,556.25	1,464.96	6,792.45
WageRate	Average wage rate of full-time employees (USD/hour)	20.22	6.29	19.38	9.74
ElecRate	Electricity rate (US cents/kilowatt-hour)	8.81	2.71	8.76	2.61
CapStock	Capital stock (million USD)	158.80	471.32	25.36	92.13
ConFilt	CWS primarily uses conventional treatment process (dummy)	0.78	0.41	0.08	0.28
DirFilt	CWS primarily uses direct filtration or disinfection only treatment process (dummy)	0.11	0.31	0.37	0.48
OthTech	CWS primarily uses membrane filtration or other treatment process (dummy)	0.11	0.32	0.54	0.50
Sold	Fraction of total production sold to other water systems	0.09	0.15	0.03	0.09
Reservoir	Primary water source is lake or reservoir (dummy)	0.56	0.50	NA	NA
RegA	CWS located in USEPA Region 1 or 2 (dummy)	0.14	0.35	0.13	0.34
RegB	CWS located in USEPA Region 3 or 4 (dummy)	0.35	0.48	0.29	0.45
RegC	CWS located in USEPA Region 5 or 6 (dummy)	0.23	0.42	0.35	0.48
RegD	CWS located in USEPA Region 7 or 8 (dummy)	0.14	0.35	0.16	0.36
RegE	CWS located in USEPA Region 9 or 10 (dummy)	0.15	0.35	0.08	0.27

*Notes:* CWS, community water system; USEPA, US Environmental Protection Agency. Descriptive statistics for surface water facilities are based on *N* = 296, except for the treatment process variables that are based on *N* = 237. Descriptive statistics for groundwater facilities are based on *N* = 200, except for the treatment process variables that are based on *N* = 156.

**Table 2.** Descriptive Statistics for Contributing Area Characteristics at Surface Water Facilities

Variable	Definition	*θ* = 1 km		*θ* = 5 km		*θ* = 10 km		*θ* = Watershed	
			
		Mean	SD	Mean	SD	Mean	SD	Mean	SD
Precip	Average annual precipitation (mm/year)	1,020.42	350.62	1,036.70	360.58	1,047.40	359.87	1,076.92	374.74
Slope	Average slope (degrees)	3.70	3.43	4.11	3.79	4.42	4.21	5.47	4.72
Forest	Fraction of land area classified as forestland	0.30	0.27	0.38	0.29	0.40	0.27	0.46	0.27
Ag	Fraction of land area classified as agriculture	0.10	0.16	0.15	0.18	0.18	0.20	0.20	0.22
Crop	Fraction of land area classified as cropland	0.04	0.11	0.06	0.13	0.08	0.16	0.09	0.18
Pasture	Fraction of land area classified as pasture	0.06	0.11	0.09	0.12	0.10	0.13	0.10	0.12
Urban	Fraction of land area classified as urban	0.21	0.24	0.20	0.21	0.17	0.18	0.10	0.11
OthLand	Fraction of land area with other classifications	0.39	0.27	0.28	0.24	0.26	0.23	0.24	0.23
CntrArea	Size of contributing area (km^2^)	2.22	1.76	32.30	23.74	93.06	68.93	40,615	261,342

*Notes*: OthLand consists of land area classified as barren, shrubland, grassland, water, and wetland. Descriptive statistics are based on *N* = 296.

**Table 3.** Descriptive Statistics for Contributing Area Characteristics in Groundwater Facilities

Variable	Definition	*θ* = 0:5 km		*θ* = 1 km		*θ* = 2:5 km		*θ* = 5 km	
			
		Mean	SD	Mean	SD	Mean	SD	Mean	SD
Precip	Average annual precipitation (mm/year)	985.21	310.85	985.59	310.35	987.18	311.28	989.25	309.20
Slope	Average slope (degrees)	1.93	1.83	2.05	1.89	2.26	2.10	2.50	2.37
Forest	Fraction of land area classified as forestland	0.20	0.22	0.22	0.22	0.24	0.23	0.26	0.23
Ag	Fraction of land area classified as agriculture	0.23	0.23	0.27	0.24	0.30	0.25	0.31	0.25
Crop	Fraction of land area classified as cropland	0.12	0.18	0.17	0.22	0.19	0.24	0.20	0.24
Pasture	Fraction of land area classified as pasture	0.10	0.15	0.10	0.14	0.11	0.13	0.11	0.12
Urban	Fraction of land area classified as urban	0.37	0.26	0.30	0.23	0.22	0.21	0.19	0.18
OthLand	Fraction of land area with other classifications	0.20	0.22	0.21	0.21	0.23	0.21	0.25	0.22
CntrArea	Size of contributing area (km^2^)	3.30	6.42	9.92	16.56	42.15	58.87	130.69	134.69

*Notes*: OthLand consists of land area classified as barren, shrubland, grassland, water, and wetland. Descriptive statistics are based on *N* = 200.

**Table 4.** Cost Function Estimates for Surface Water Facilities

	*θ* = 5 km		*θ* = 10 km		*θ* = Watershed	
		
	Model 1	Model 2	Model 1	Model 2	Model 1	Model 2
ln(WatVol)	0.996*** (0.078)	0.996*** (0.078)	1.004*** (0.080)	1.004*** (0.080)	±1.027*** (0.081)	±1.027*** (0.081)
ln(WageRate)	0.663*** (0.169)	0.668*** (0.170)	0.634*** (0.172)	0.639*** (0.175)	0.638*** (0.186)	0.632*** (0.188)
ln(ElecRate)	0.337** (0.169)	0.332* (0.170)	0.366** (0.172)	0.361** (0.175)	0.362* (0.186)	0.368* (0.188)
ln(CapStock)	−0.195** (0.076)	−0.194** (0.075)	−0.204*** (0.078)	−0.204*** (0.077)	−0.235*** (0.079)	−0.234*** (0.079)
ln(Sold+l)	−0.773** (0.367)	−0.777**(0.369)	0.785** (0.375)	−0.788** (0.377)	−0.660* (0.372)	−0.657* (0.371)
Reservoir	0.098 (0.103)	0.093 (0.106)	0.104 (0.104)	0.102 (0.108)	0.135 (0.101)	0.138 (0.102)
ln(Precip)	0.299** (0.147)	0.294** (0.147)	0.266* (0.159)	0.261 (0.160)	0.054 (0.229)	−0.054 (0.231)
ln(Slope)	0.145** (0.066)	0.136** (0.069)	0.125* (0.066)	0.117* (0.068)	−0.057 (0.085)	−0.060 (0.084)
ln(OthLand+l)	0.613 (0.420)	0.579 (0.423)	0.519 (0.420)	0.490 (0.420)	0.053 (0.497)	0.036 (0.492)
ln(Ag+l)	0.440 (0.424)		0.370 (0.409)		0.112 (0.403)	
ln(Crop+l)		0.291 (0.673)		0.283 (0.546)		0.127 (0.478)
ln(Pasture+l)		0.444 (0.481)		0.320 (0.491)		−0.036 (0.508)
ln(Urban+l)	0.783** (0.369)	0.747** (0.376)	0.930** (0.387)	0.900** (0.391)	0.930 (0.582)	0.923 (0.574)
Constant	4.227*** (1.256)	4.275*** (1.258)	4.572*** (1.344)	4.628*** (1.352)	7.359*** (1.830)	7.371*** (1.847)
Reg FE	Yes	Yes	Yes	Yes	Yes	Yes
*R*^2^	0.882	0.882	0.881	0.881	0.879	0.879
*N*	296	296	296	296	296	296

*Notes*: Standard errors, reported in parentheses, are estimated using a bootstrap procedure with 1,000 replications.

* *p* < 0.1

** *p* < 0.05

*** *p* < 0.01.

**Table 5.** Land Use Elasticities of Cost for Surface Water Facilities

	*θ* = 5km	*θ* = 10km	*θ* = Watershed
Ag	0.057	0.056	0.018
	(0.055)	(0.062)	(0.066)
Crop	0.016	0.021	0.011
	(0.038)	(0.040)	(0.041)
Pasture	0.036	0.029	0.003
	(0.039)	(0.044)	(0.048)
Urban	0.124**	0.129**	0.082^†^
	(0.063)	(0.056)	(0.051)

*Notes*: Estimates for Ag based on Model 1. Estimates for Crop, Pasture, and Urban based on Model 2. Standard errors reported in parentheses.

† *p* < 0.15

* *p* < 0.1

** *p* < 0.05

*** *p* < 0.01.

**Table 6.** Cost Function Estimates for Groundwater Facilities

	*θ* = 0.5 km		*θ* = 1 km		*θ* = 2.5 km	
		
	Model 1	Model 2	Model 1	Model 2	Model 1	Model 2
ln(WatVol)	0.912*** (0.060)	0.915*** (0.058)	0.916*** (0.065)	0.919*** (0.060)	0.907*** (0.064)	0.910*** (0.065)
ln(WageRate)	0.132 (0.198)	0.139 (0.191)	0.155 (0.196)	0.160 (0.199)	0.113 (0.208)	0.115 (0.198)
ln(ElecRate)	0.868*** (0.198)	0.861*** (0.191)	0.845*** (0.196)	0.840*** (0.199)	0.887*** (0.208)	0.885*** (0.198)
ln(CapStock)	0.007 (0.064)	−0.000 (0.063)	0.019 (0.066)	0.012 (0.061)	0.017 (0.064)	0.012 (0.065)
ln(Sold+l)	−1.536 (1.271)	−1.469 (1.218)	−1.607 (1.166)	−1.515 (1.218)	−1.346 (1.091)	−1.266 (1.243)
ln(Precip)	0.282 (0.302)	0.265 (0.313)	0.321 (0.300)	0.287 (0.326)	0.289 (0.314)	0.268 (0.356)
ln(Slope)	0.162** (0.080)	0.141* (0.081)	0.187** (0.084)	0.161* (0.086)	0.181* (0.094)	0.162* (0.098)
ln(OthLand+l)	0.741 (0.563)	0.731 (0.576)	0.846 (0.637)	0.840 (0.607)	0.565 (0.645)	0.605 (0.672)
ln(Ag+l)	1.203** (0.583)		1.137* (0.608)		0.797 (0.593)	
ln(Crop+l)		0.898 (0.635)		0.905 (0.576)		0.657 (0.614)
ln(Pasture+l)		1.495** (0.674)		1.522** (0.694)		1.158 (0.728)
ln(Urban+l)	0.117 (0.512)	0.122 (0.504)	−0.078 (0.650)	−0.072 (0.639)	0.011 (0.739)	0.014 (0.732)
Constant	2.763 (2.123)	2.946 (2.227)	2.353 (2.200)	2.651 (2.334)	2.660 (2.307)	2.839 (2.585)
Reg FE	Yes	Yes	Yes	Yes	Yes	Yes
*R*^2^	0.892	0.893	0.892	0.893	0.890	0.890
*N*	200	200	200	200	200	200

*Notes*: Standard errors, reported in parentheses, are estimated using a bootstrap procedure with 1,000 replications.

* *p* < 0.1

** *p* < 0.05

*** *p* < 0.01.

**Table 7.** Land Use Elasticities of Cost for Groundwater Facilities

	*θ* = 0.5km	*θ* = 1km	*θ* = 2.5km
Ag	0.224**	0.241*	0.184
	(0.108)	(0.129)	(0.137)
Crop	0.099	0.128^†^	0.106
	(0.070)	(0.082)	(0.099)
Pasture	0.142**	0.143**	0.111^†^
	(0.064)	(0.065)	(0.070)
Urban	0.033	−0.017	0.003
	(0.136)	(0.146)	(0.134)

*Notes*: Estimates for Ag based on Model 1. Estimates for Crop, Pasture, and Urban based on Model 2.

Standard errors reported in parentheses.

† *p* < 0.15

* *p* < 0.1

** *p* < 0.05

*** *p* < 0.01.

## Footnotes

1 Price and Heberling (2018) review the literature statistically linking water treatment costs to source water quality and report elasticities of cost (i.e., the percentage change in the treatment costs given a 1% change in the water quality measure) for key results. For comparability, we utilize these elasticities when describing results from existing studies.

2 CWSs supply potable water through pipes or other constructed conveyances to at least 15 service connections used by year-round residents or regularly serve at least 25 year-round residents (40 C.F.R. § 141.2 2016).

3 This constraint affects the relative magnitude of coefficients in vector *β*_*ph*_, but it has little effect on other parameter values. For models estimated without the constraint, Wald tests indicate that the sum of *β*_*ph*_ is not significantly different from one.

4 We tested other constants with the land use variables, and they had a negligible effect on estimated elasticities.

5 A publicly available version of the CWSS data, which does not contain CWS identifiers, can be downloaded at https://www.epa.gov/dwreginfo/community-water-system-survey.

6 Fourteen outliers were removed from the surface water dataset and nine from the groundwater dataset. Outliers were defined as wage rates greater than $150 per hour and production output greater than 162,754 million gallons (equivalent to a logged output of 12). The latter criterion was based on a scatter plot of logged costs and logged output, which showed CWSs in the sample with output greater than 162,754 million gallons had unrealistically low per unit costs.

7 We use the 1.52% depreciation rate reported in Fraumeni (1997) for government water systems. The weighted average cost of debt and equality calculation takes the form *γ* = [(1 – *d* × *r*_*e)*_ + (*d* × *r*_*d*_)], where *d* is the proportion of capital expenditures funded by debt, *r*_*e*_ is the cost of equity, and *s* is the cost of debt. The municipal bond rate serves as the cost of debt (Bloomberg Barclays 2016). The cost of equity calculation takes the form *r*_*e*_ *= r*_*f*_ *+ δ × r*_*p*_, where *r*_*f*_ is the risk-free return as measured by the average return on 10-year treasury bills, *r*_*p*_ the risk premium (i.e., the difference between market and risk-free returns), and *δ* the degree of industry equity-specific risk. Values for these terms are obtained from Damodaran (2018a, 2018b).

8 Spatial analyses are performed in ArcMap 10.3.

9 All cost functions were estimated in Stata 15.

10 A reviewer suggested leaving out agriculture and including forestland in the models. We ran these alternate models and they are available upon request. Results show that forest and urban land use, relative to agriculture, are significant and negatively correlated with costs for groundwater models at the 0.5 km and 1 km contributing areas. Forestland was not significant in the surface water models.

11 As a spatial reference, 0.064, 0.144, 0.008, and 0.027 km^2^ are roughly equivalent to the size of 9, 22, 1, and 4 standard sized football pitches, respectively.
