# Supplementary material for: The Effects of Agricultural and Urban Land Use on Drinking Water Treatment Costs: An Analysis of United States Community Water Systems
Source: Water Econ Policy. Author manuscript; Available in PMC 2021 Aug 5. (PMC8340599; doi:10.1142/s2382624x20500083)
Supplement: 1 [file NIHMS1651760-supplement-1.pdf]

Water Econs. Policy 2020.06. Downloaded from www.worldscientific.com
by 134.67.29.68 on 06/01/21. Re-use and distribution is strictly not permitted, except for Open Access articles.

Appendix

Table S1. Observations by Dataset and Source

	Surface Water Dataset	Groundwater Dataset	Total
CWSs that completed the CWSS only	136	178	314
CWSs that completed the WTPQ only	103	17	120
CWSs that completed the CWSS and WTPQ	57	5	62
Total	296	200	496

Notes: CWS, community water system; CWSS, 2006 Community Water System Survey; WTPQ, Water Treatment Plant Questionnaire. Data from these surveys were merged with information about land use, land slope, and average annual precipitation, which were derived from the USEPA Office of Water's Spatial Database of public water supply intakes and wells, National Land Cover Database (NLCD) 2006, Parameter-Elevation Regression on Independent Slopes (PRISM), and National Elevation Dataset.

Water Econs. Policy 2020.06. Downloaded from www.worldscientific.com
by 134.67.29.68 on 06/01/21. Re-use and distribution is strictly not permitted, except for Open Access articles.

Table S2. Percentage of Community Water System by Population Served

Population Category	Surface Water Facilities		Groundwater Facilities	
	Sample Data (%)	CWSS Data (%)	Sample Data (%)	CWSS Data (%)
Population < 101	1.0	7.8	14.0	28.6
Population 101–500	8.4	14.0	23.0	34.2
Population 501–3,300	10.5	24.5	29.0	24.2
Population 3,301–10,000	9.8	19.8	6.5	7.3
Population 10,001–50,000	26.0	21.2	17.5	4.8
Population 50,001–100,000	17.9	5.3	5.5	0.6
Population 100,001–500,000	22.6	6.1	3.5	0.3
Population $> 500,000$	3.7	1.4	1.0	0.04
Total	100	100	100	100

Note: The CWSS data are based on information available in USEPA (2009) and pertain to public and private CWSs.

Table S3. Average 2006 Production by Population Served (Million Gallons)

Population Category	Surface Water Facilities		Groundwater Facilities	
	Sample Data	CWSS Data	Sample Data	CWSS Data
Population < 101	1.78	—	2.29	—
Population 101–500	15.00	9.86	10.21	10.56
Population 501–3,300	213.94	110.96	67.79	106.58
Population 3,301–10,000	533.21	364.27	304.94	492.39
Population 10,001–50,000	1,939.96	1,292.10	1,203.12	2,067.36
Population 50,001–100,000	4,331.12	5,478.29	4,356.40	3,323.69
Population 100,001–500,000	14,283.51	12,313.28	9,984.23	14,459.11
Population $> 500,000$	64,540.84	71,694.03	62,321.85	60,569.93
Total	6,987.65	3,980.42	1,464.96	1,728.64

Notes: CWSS data are based on information available in USEPA (2009) and pertain to public and private CWSs, which are defined as water systems that supply potable water to at least 15 service connections used by year-round residents or regularly serve at least 25 year-round residents.

Table S4. Cost Function Estimates for Surface Water Facilities (Model Specification 1)

	$\theta = 1\,\text{km}$	$\theta = 5\,\text{km}$	$\theta = 10\,\text{km}$	$\theta = \text{Watershed}$
ln (WatVol)	1.006*** (0.080)	0.996*** (0.078)	1.004*** (0.080)	1.027*** (0.081)
ln (WageRate)	0.694*** (0.170)	0.663*** (0.169)	0.634*** (0.172)	0.638*** (0.186)
ln (ElecRate)	0.306* (0.170)	0.337** (0.169)	0.366** (0.172)	0.362* (0.186)
ln(CapStock)	−0.202*** (0.075)	−0.195** (0.076)	−0.204*** (0.078)	−0.235*** (0.079)
ln(Sold+1)	−0.759** (0.368)	−0.773** (0.367)	−0.785** (0.375)	−0.660* (0.372)

Water Econs. Policy 2020.06. Downloaded from www.worldscientific.com
by 134.67.29.68 on 06/01/21. Re-use and distribution is strictly not permitted, except for Open Access articles.

Table S4. (*Continued*)

	$\theta = 1\,\text{km}$	$\theta = 5\,\text{km}$	$\theta = 10\,\text{km}$	$\theta =$ Watershed
Reservoir	0.074 (0.105)	0.098 (0.103)	0.104 (0.104)	0.135 (0.101)
RegB	0.491*** (0.170)	0.482*** (0.174)	0.493*** (0.176)	0.530*** (0.178)
RegC	0.567*** (0.168)	0.638*** (0.172)	0.626*** (0.174)	0.467*** (0.181)
RegD	0.664*** (0.246)	0.644*** (0.241)	0.638** (0.251)	0.598** (0.284)
RegE	0.468* (0.266)	0.375 (0.264)	0.378 (0.264)	0.552** (0.275)
ln(Precip)	0.274** (0.137)	0.299** (0.147)	0.266* (0.159)	−0.054 (0.229)
ln(Slope)	0.036 (0.055)	0.145** (0.066)	0.125* (0.066)	−0.057 (0.085)
ln (OthLand+1)	0.413 (0.453)	0.613 (0.420)	0.519 (0.420)	0.053 (0.497)
ln (Ag+1)	−0.011 (0.431)	0.440 (0.424)	0.370 (0.409)	0.112 (0.403)
ln (Urban+1)	0.255 (0.375)	0.783** (0.369)	0.930** (0.387)	0.930 (0.582)
Constant	4.682*** (1.168)	4.227*** (1.256)	4.572*** (1.344)	7.359*** (1.830)
R^2	0.880	0.882	0.881	0.879
N	296	296	296	296

Notes: Standard errors, reported in parentheses, are estimated using a bootstrap procedure with 1,000 replications. $^{*}p < 0.1$, $^{**}p < 0.05$, $^{***}p < 0.01$.

Table S5. Cost Function Estimates for Surface Water Facilities (Model Specification 2)

	$\theta = 1\,\text{km}$	$\theta = 5\,\text{km}$	$\theta = 10\,\text{km}$	$\theta =$ Watershed
ln (WatVol)	1.005*** (0.079)	0.996*** (0.078)	1.004*** (0.080)	1.027*** (0.081)
ln (WageRate)	0.702*** (0.172)	0.668*** (0.170)	0.639*** (0.175)	0.632*** (0.188)
ln (ElecRate)	0.298* (0.172)	0.332* (0.170)	0.361** (0.175)	0.368* (0.188)
ln(CapStock)	−0.197*** (0.075)	−0.194** (0.075)	−0.204*** (0.077)	−0.234*** (0.079)
ln(Sold+1)	−0.752** (0.366)	−0.777** (0.369)	−0.788** (0.377)	−0.657* (0.371)
Reservoir	0.064 (0.107)	0.093 (0.106)	0.102 (0.108)	0.138 (0.102)
RegB	0.475*** (0.170)	0.480*** (0.169)	0.494*** (0.173)	0.543*** (0.172)
RegC	0.574*** (0.169)	0.639*** (0.173)	0.628*** (0.175)	0.470*** (0.182)
RegD	0.656*** (0.246)	0.648*** (0.244)	0.639** (0.252)	0.601** (0.286)
RegE	0.484* (0.266)	0.383 (0.270)	0.383 (0.269)	0.553** (0.278)
ln(Precip)	0.262* (0.136)	0.294** (0.147)	0.261 (0.160)	−0.054 (0.231)
ln(Slope)	0.030 (0.056)	0.136** (0.069)	0.117* (0.068)	−0.060 (0.084)
ln (OthLand+1)	0.391 (0.457)	0.579 (0.423)	0.490 (0.420)	0.036 (0.492)
ln (Crop+1)	−0.553 (0.570)	0.291 (0.673)	0.283 (0.546)	0.127 (0.478)
ln(Pasture+1)	0.479 (0.496)	0.444 (0.481)	0.320 (0.491)	−0.036 (0.508)
ln (Urban+1)	0.232 (0.376)	0.747** (0.376)	0.900** (0.391)	0.923 (0.574)
Constant	4.718*** (1.163)	4.275*** (1.258)	4.628*** (1.352)	7.371*** (1.847)
R^2	0.881	0.882	0.881	0.879
N	296	296	296	296

Notes: Standard errors, reported in parentheses, are estimated using a bootstrap procedure with 1,000 replications. $^{*}p < 0.1$, $^{**}p < 0.05$, $^{***}p < 0.01$.

Water Econs. Policy 2020.06. Downloaded from www.worldscientific.com
by 134.67.29.68 on 06/01/21. Re-use and distribution is strictly not permitted, except for Open Access articles.

Table S6. Cost Function Estimates for Surface Water Facilities (Model Specification 3)

	$\theta = 1$ km	$\theta = 5$ km	$\theta = 10$ km	$\theta =$ Watershed
ln (WatVol)	0.972*** (0.095)	0.968*** (0.092)	0.971*** (0.093)	0.999*** (0.096)
ln (WageRate)	0.557*** (0.215)	0.527** (0.212)	0.497** (0.207)	0.450** (0.223)
ln (ElecRate)	0.443** (0.215)	0.473** (0.212)	0.503** (0.207)	0.550** (0.223)
ln(CapStock)	−0.176** (0.089)	−0.172* (0.090)	−0.178* (0.092)	−0.213** (0.096)
DirFilt	−0.149 (0.216)	−0.151 (0.217)	−0.168 (0.218)	−0.237 (0.223)
OthTech	−0.163 (0.212)	−0.126 (0.212)	−0.152 (0.216)	−0.192 (0.221)
ln(Sold+1)	−0.832** (0.401)	−0.846** (0.411)	−0.859** (0.411)	−0.765* (0.399)
Reservoir	0.071 (0.127)	0.091 (0.125)	0.098 (0.128)	0.122 (0.126)
RegB	0.569** (0.227)	0.548** (0.236)	0.553** (0.239)	0.592** (0.239)
RegC	0.616*** (0.216)	0.675*** (0.227)	0.657*** (0.229)	0.469* (0.247)
RegD	0.845*** (0.308)	0.825*** (0.304)	0.827*** (0.313)	0.783** (0.357)
RegE	0.667* (0.356)	0.572 (0.364)	0.592 (0.360)	0.773** (0.360)
ln(Precip)	0.261 (0.159)	0.292* (0.172)	0.248 (0.183)	−0.086 (0.258)
ln(Slope)	−0.016 (0.072)	0.083 (0.090)	0.059 (0.091)	−0.124 (0.104)
ln(OthLand+1)	0.128 (0.571)	0.346 (0.516)	0.199 (0.488)	−0.219 (0.576)
ln(Ag+1)	−0.310 (0.494)	0.289 (0.499)	0.186 (0.509)	−0.052 (0.513)
ln (Urban+1)	0.162 (0.410)	0.617 (0.438)	0.722 (0.469)	0.747 (0.821)
Constant	4.882*** (1.356)	4.400*** (1.484)	4.836*** (1.570)	7.805*** (2.101)
R^2	0.872	0.873	0.872	0.871
N	237	237	237	237

Standard errors, reported in parentheses, are estimated using a bootstrap procedure with 1,000 replications.
$*p < 0.1$, $**p < 0.05$, $***p < 0.01$.

Table S7. Cost Function Estimates for Surface Water Facilities (Model Specification 4)

	$\theta = 1$ km	$\theta = 5$ km	$\theta = 10$ km	$\theta =$ Watershed
ln (WatVol)	0.975*** (0.094)	0.967*** (0.092)	0.970*** (0.094)	0.994*** (0.098)
ln (WageRate)	0.562*** (0.218)	0.526** (0.212)	0.489** (0.209)	0.423* (0.224)
ln (ElecRate)	0.438** (0.218)	0.474** (0.212)	0.511** (0.209)	0.577** (0.224)
ln(CapStock)	−0.175** (0.089)	−0.172* (0.089)	−0.178* (0.092)	−0.211** (0.096)
DirFilt	−0.140 (0.215)	−0.155 (0.216)	−0.176 (0.217)	−0.251 (0.223)
OthTech	−0.147 (0.206)	−0.133 (0.212)	−0.162 (0.215)	−0.205 (0.220)
ln(Sold+1)	−0.823** (0.394)	−0.844** (0.402)	−0.853** (0.413)	−0.750* (0.401)
Reservoir	0.067 (0.128)	0.093 (0.125)	0.107 (0.129)	0.139 (0.126)
RegB	0.556** (0.228)	0.554** (0.231)	0.570** (0.236)	0.638*** (0.237)
RegC	0.622*** (0.217)	0.675*** (0.227)	0.660*** (0.229)	0.484* (0.249)
RegD	0.836*** (0.308)	0.828*** (0.304)	0.836*** (0.313)	0.806** (0.358)
RegE	0.666* (0.356)	0.576 (0.366)	0.598* (0.361)	0.783** (0.360)
ln(Precip)	0.255 (0.158)	0.289* (0.173)	0.248 (0.184)	−0.075 (0.260)
ln(Slope)	−0.018 (0.073)	0.079 (0.089)	0.058 (0.089)	−0.123 (0.103)
ln(OthLand+1)	0.116 (0.576)	0.322 (0.519)	0.182 (0.487)	−0.213 (0.576)

Table S7. (*Continued*)

	$\theta = 1\,\text{km}$	$\theta = 5\,\text{km}$	$\theta = 10\,\text{km}$	$\theta = \text{Watershed}$
ln (Crop+1)	−0.613 (0.607)	0.265 (0.742)	0.237 (0.625)	0.114 (0.579)
ln(Pasture+1)	0.062 (0.617)	0.176 (0.578)	−0.007 (0.585)	−0.399 (0.637)
ln (Urban+1)	0.157 (0.410)	0.595 (0.435)	0.718 (0.460)	0.818 (0.794)
Constant	4.892*** (1.359)	4.442*** (1.488)	4.860*** (1.579)	7.733***(2.126)
R^2	0.873	0.873	0.873	0.872
N	237	237	237	237

Notes: Standard errors, reported in parentheses, are estimated using a bootstrap procedure with 1,000 replications. *$p < 0.1$, **$p < 0.05$, ***$p < 0.01$.

Table S8. Cost Function Estimates for Groundwater Facilities (Model Specification 1)

	$\theta = 0.5\,\text{km}$	$\theta = 1\,\text{km}$	$\theta = 2.5\,\text{km}$	$\theta = 5\,\text{km}$
ln (WatVol)	0.912*** (0.060)	0.916*** (0.065)	0.907*** (0.064)	0.904*** (0.061)
ln (WageRate)	0.132 (0.198)	0.155 (0.196)	0.113 (0.208)	0.082 (0.197)
ln (ElecRate)	0.868*** (0.198)	0.845*** (0.196)	0.887*** (0.208)	0.918*** (0.197)
ln (CapStock)	0.007 (0.064)	0.019 (0.066)	0.017 (0.064)	0.012 (0.065)
ln(Sold+1)	−1.536 (1.271)	−1.607 (1.166)	−1.346 (1.091)	−1.241 (1.163)
RegB	0.542** (0.213)	0.520** (0.212)	0.602*** (0.230)	0.669*** (0.227)
RegC	0.711*** (0.222)	0.698*** (0.220)	0.744*** (0.238)	0.776*** (0.226)
RegD	0.693** (0.284)	0.658** (0.281)	0.727** (0.291)	0.774*** (0.276)
RegE	0.297 (0.463)	0.301 (0.467)	0.255 (0.501)	0.191 (0.483)
ln (Precip)	0.282 (0.302)	0.321 (0.300)	0.289 (0.314)	0.274 (0.343)
ln (Slope)	0.162** (0.080)	0.187** (0.084)	0.181* (0.094)	0.200* (0.103)
ln (OthLand+1)	0.741 (0.563)	0.846 (0.637)	0.565 (0.645)	0.462 (0.687)
ln (Ag+1)	1.203** (0.583)	1.137* (0.608)	0.797 (0.593)	0.624 (0.591)
ln (Urban+1)	0.117 (0.512)	−0.078 (0.658)	0.011 (0.739)	0.136 (0.739)
Constant	2.763 (2.123)	2.353 (2.200)	2.660 (2.307)	2.827 (2.507)
R^2	0.892	0.892	0.890	0.890
N	200	200	200	200

Notes: Standard errors, reported in parentheses, are estimated using a bootstrap procedure with 1,000 replications. *$p < 0.1$, **$p < 0.05$, ***$p < 0.01$.

Water Econs. Policy 2020.06. Downloaded from www.worldscientific.com
by 134.67.29.68 on 06/01/21. Re-use and distribution is strictly not permitted, except for Open Access articles.

Water Econs. Policy 2020.06. Downloaded from www.worldscientific.com
by 134.67.29.68 on 06/01/21. Re-use and distribution is strictly not permitted, except for Open Access articles.

Table S9. Cost Function Estimates for Groundwater Facilities (Model Specification 2)

	$\theta = 0.5$ km	$\theta = 1$ km	$\theta = 2.5$ km	$\theta = 5$ km
ln (WatVol)	0.915*** (0.058)	0.919*** (0.060)	0.910*** (0.065)	0.906*** (0.062)
ln (WageRate)	0.139 (0.191)	0.160 (0.199)	0.115 (0.198)	0.082 (0.197)
ln (ElecRate)	0.861*** (0.191)	0.840*** (0.199)	0.885*** (0.198)	0.918*** (0.197)
ln (CapStock)	−0.000 (0.063)	0.012 (0.061)	0.012 (0.065)	0.009 (0.063)
ln (Sold+1)	−1.469 (1.218)	−1.515 (1.218)	−1.266 (1.243)	−1.217 (1.198)
RegB	0.506** (0.209)	0.474** (0.216)	0.561** (0.219)	0.653*** (0.216)
RegC	0.704*** (0.228)	0.684*** (0.221)	0.730*** (0.231)	0.770*** (0.222)
RegD	0.670** (0.287)	0.619** (0.287)	0.693** (0.301)	0.764*** (0.290)
RegE	0.311 (0.473)	0.302 (0.470)	0.260 (0.492)	0.196 (0.476)
ln (Precip)	0.265 (0.313)	0.287 (0.326)	0.268 (0.356)	0.263 (0.344)
ln (Slope)	0.141* (0.081)	0.161* (0.086)	0.162* (0.098)	0.190* (0.102)
ln (OthLand+1)	0.731 (0.576)	0.840 (0.607)	0.605 (0.672)	0.462 (0.673)
ln (Crop+1)	0.898 (0.635)	0.905 (0.576)	0.657 (0.614)	0.550 (0.556)
ln (Pasture+1)	1.495** (0.674)	1.522** (0.694)	1.158 (0.728)	0.731 (0.735)
ln (Urban+1)	0.122 (0.504)	−0.072 (0.639)	0.014 (0.732)	0.126 (0.725)
Constant	2.946 (2.227)	2.651 (2.334)	2.839 (2.585)	2.923 (2.505)
R^2	0.893	0.892	0.890	0.890
N	200	200	200	200

Notes: Standard errors, reported in parentheses, are estimated using a bootstrap procedure with 1,000 replications. $^*p < 0.1$, $^{**}p < 0.05$, $^{***}p < 0.01$.

Table S10. Cost Function Estimates for Groundwater Facilities (Model Specification 3)

	$\theta = 0.5$ km	$\theta = 1$ km	$\theta = 2.5$ km	$\theta = 5$ km
ln (WatVol)	0.885*** (0.080)	0.893*** (0.077)	0.886*** (0.081)	0.883*** (0.082)
ln (WageRate)	0.073 (0.227)	0.110 (0.223)	0.090 (0.225)	0.066 (0.224)
ln (ElecRate)	0.927*** (0.227)	0.890*** (0.223)	0.910*** (0.225)	0.934*** (0.224)
ln (CapStock)	0.037 (0.084)	0.053 (0.080)	0.052 (0.084)	0.041 (0.084)
DirFilt	0.152 (0.397)	0.125 (0.400)	0.080 (0.394)	0.084 (0.366)
OthTech	0.194 (0.412)	0.197 (0.411)	0.173 (0.409)	0.176 (0.384)
ln(Sold+1)	−1.201 (1.579)	−1.335 (1.582)	−0.972 (1.596)	−0.754 (1.551)
RegB	0.521** (0.226)	0.498** (0.227)	0.568*** (0.217)	0.637*** (0.233)
RegC	0.644** (0.267)	0.645** (0.253)	0.709*** (0.244)	0.747*** (0.254)
RegD	0.614 (0.379)	0.605 (0.374)	0.641* (0.364)	0.671* (0.368)
RegE	0.269 (0.754)	0.313 (0.703)	0.279 (0.707)	0.183 (0.743)
ln (Precip)	0.006 (0.321)	0.033 (0.323)	0.091 (0.364)	0.094 (0.394)
ln (Slope)	0.129 (0.129)	0.140 (0.131)	0.197 (0.145)	0.239 (0.149)
ln (OthLand+1)	0.925 (0.724)	0.858 (0.830)	1.036 (0.936)	1.039 (0.972)
ln (Ag+1)	1.247* (0.719)	1.096 (0.787)	1.006 (0.777)	0.849 (0.778)
ln(Urban+1)	0.084 (0.706)	−0.412 (0.851)	−0.224 (0.931)	−0.040 (0.910)
Constant	4.407* (2.379)	4.130* (2.483)	3.618 (2.804)	3.638 (3.024)
R^2	0.885	0.885	0.883	0.883
N	156	156	156	156

Notes: Standard errors, reported in parentheses, are estimated using a bootstrap procedure with 1,000 replications. $^*p < 0.1$, $^{**}p < 0.05$, $^{***}p < 0.01$.

Water Econs. Policy 2020.06. Downloaded from www.worldscientific.com by 134.67.29.68 on 06/01/21. Re-use and distribution is strictly not permitted, except for Open Access articles.

Table S11. Cost Function Estimates for Groundwater Facilities (Model Specification 4)

	$\theta = 0.5$ km	$\theta = 1$ km	$\theta = 2.5$ km	$\theta = 5$ km
ln (WatVol)	0.889*** (0.078)	0.897*** (0.082)	0.890*** (0.079)	0.885*** (0.079)
ln (WageRate)	0.074 (0.222)	0.105 (0.222)	0.076 (0.228)	0.063 (0.217)
ln (ElecRate)	0.926*** (0.222)	0.895*** (0.222)	0.924*** (0.228)	0.937*** (0.217)
ln (CapStock)	0.029 (0.083)	0.045 (0.081)	0.043 (0.081)	0.039 (0.084)
DirFilt	0.144 (0.403)	0.121 (0.383)	0.082 (0.387)	0.089 (0.385)
OthTech	0.177 (0.419)	0.181 (0.395)	0.170 (0.404)	0.177 (0.396)
ln(Sold+1)	−1.121 (1.683)	−1.203 (1.529)	−0.807 (1.515)	−0.718 (1.478)
RegB	0.502** (0.225)	0.467** (0.220)	0.532** (0.226)	0.627*** (0.240)
RegC	0.645** (0.260)	0.645** (0.255)	0.713*** (0.247)	0.746*** (0.260)
RegD	0.601 (0.386)	0.576 (0.378)	0.620* (0.375)	0.667* (0.375)
RegE	0.278 (0.766)	0.323 (0.709)	0.309 (0.728)	0.190 (0.733)
ln (Precip)	−0.005 (0.330)	0.012 (0.332)	0.077 (0.359)	0.083 (0.394)
ln (Slope)	0.115 (0.133)	0.119 (0.136)	0.173 (0.151)	0.229 (0.162)
ln (OthLand+1)	0.911 (0.717)	0.863 (0.787)	1.097 (0.885)	1.022 (0.957)
ln (Crop+1)	0.999 (0.827)	0.882 (0.779)	0.823 (0.751)	0.761 (0.751)
ln (Pasture+1)	1.406* (0.787)	1.411* (0.817)	1.449 (0.906)	0.884 (0.922)
ln(Urban+1)	0.086 (0.704)	−0.391 (0.799)	−0.198(0.962)	−0.061 (0.904)
Constant	4.562* (2.448)	4.355* (2.526)	3.767 (2.785)	3.746 (3.005)
R^2	0.885	0.885	0.884	0.883
N	156	156	156	156

Notes: Standard errors, reported in parentheses, are estimated using a bootstrap procedure with 1,000 replications. $^*p < 0.1$, $^{**}p < 0.05$, $^{***}p < 0.01$.

Table S12. Avoided Costs Per Square Kilometer of Preserved Forestland

	Surface Water Facilities			Groundwater Facilities		
	$\theta = 5$ km	$\theta = 10$ km	$\theta =$ Watershed	$\theta = 0.5$ km	$\theta = 1$ km	$\theta = 2.5$ km
Avoided costs ($)	12,662	13,173	8,374	4,150	4,465	3,409
Preserved forestland (km^2)	0.064	0.155	39.706	0.008	0.027	0.126
Avoided costs/km^2 of preserved forestland	196,618	84,867	211	550,706	167,263	27,016

Notes: Avoided costs are the average savings that result from preventing the displacement of forestland equivalent to a 1% increase in urban land (surface water systems) or agricultural land (groundwater systems). They are calculated as the product of the estimated elasticity and average variable costs. Land area, calculated as 1% of the product of the average contributing area and average fraction of urban (surface water systems) or agricultural land (groundwater systems), represents the average amount of forestland that would be preserved to achieve the above avoided costs. Avoided costs and avoided costs per km^2 of preserved forestland can be calculated for a specific CWS given information on annual treatment costs and forestland with the contributing area.
